# Programme 18th Annual Convention T. S M. A.

**Published:** 1886-04

**Authors:** H. K. Leake

**Affiliations:** Chairman Committee on Programme


### PROGRAMME

*Of the Eighteenth Annual Meeting of the Texas State Medical Association, at Dallas, April 27, 28, 29 and 30, 1886.*

April 27th—Address of welcome by Hon. John Henry Brown, Mayor of Dallas, at 10:30 a. m.

Immediately thereafter an address by Dr. S. D. Thruston, in behalf of the Dallas County Medical Society, followed by Col. W. L. Crawford, for the citizens at large. Reply by the President of the State Medical Society, and opening of the Society with prayer by Rt. Rev. Bishop A. C. Garrett.

The Society, in a body, will attend the opera, at 8 p. m.

April 28th—Address of the President, in Merchants' Exchange, at 8 p. m. Banquet at 10 p. m.

April 29th—Address of Dr. A. G. Clopton, essayist, at Merchants' Exchange, at 8 p. m. Ball at opera-house, at 10 p. m.

April 30th—Homes of resident physicians open from 3 p. m. to 9 p. m., to the members of the Association.

H. K. LEAKE,
Chairman Committee on Programme.

We are requested by Chairman Chilton, of the Arrangement

Committee to state that the Grand Windsor and the St. George Hotels will give reduced rates to members of the association, during the meeting.

